# Classifying migraine using PET compressive big data analytics of brain’s *μ*-opioid and D2/D3 dopamine neurotransmission

**DOI:** 10.3389/fphar.2023.1173596

**Published:** 2023-06-13

**Authors:** Simeone Marino, Hassan Jassar, Dajung J. Kim, Manyoel Lim, Thiago D. Nascimento, Ivo D. Dinov, Robert A. Koeppe, Alexandre F. DaSilva

**Affiliations:** ^1^ Statistics Online Computational Resource, Department of Health Behavior and Biological Sciences, University of Michigan, Ann Arbor, MI, United States; ^2^ Department of Microbiology and Immunology, University of Michigan, Ann Arbor, MI, United States; ^3^ The Michigan Neuroscience Institute (MNI), University of Michigan, Ann Arbor, MI, United States; ^4^ Headache and Orofacial Pain Effort (H.O.P.E.) Laboratory, Department of Biologic and Materials Sciences and Prosthodontics, University of Michigan School of Dentistry, Ann Arbor, MI, United States; ^5^ Department of Computational Medicine and Bioinformatics, University of Michigan, Ann Arbor, MI, United States; ^6^ Michigan Institute for Data Science, University of Michigan, Ann Arbor, MI, United States; ^7^ Department of Radiology, Division of Nuclear Medicine, University of Michigan Medical School, Ann Arbor, MI, United States

**Keywords:** migraine disease, artificial intelligence, dopamine (raclopride), *μ*-opioid (carfentanil), computer-aided diagnosis, PET imaging data, CBDA

## Abstract

**Introduction:** Migraine is a common and debilitating pain disorder associated with dysfunction of the central nervous system. Advanced magnetic resonance imaging (MRI) studies have reported relevant pathophysiologic states in migraine. However, its molecular mechanistic processes are still poorly understood *in vivo*. This study examined migraine patients with a novel machine learning (ML) method based on their central *μ*-opioid and dopamine D2/D3 profiles, the most critical neurotransmitters in the brain for pain perception and its cognitive-motivational interface.

**Methods:** We employed compressive Big Data Analytics (CBDA) to identify migraineurs and healthy controls (HC) in a large positron emission tomography (PET) dataset. 198 PET volumes were obtained from 38 migraineurs and 23 HC during rest and thermal pain challenge. 61 subjects were scanned with the selective *μ*-opioid receptor (μOR) radiotracer [^11^C]Carfentanil, and 22 with the selective dopamine D2/D3 receptor (DOR) radiotracer [^11^C]Raclopride. PET scans were recast into a 1D array of 510,340 voxels with spatial and intensity filtering of non-displaceable binding potential (BP_ND_), representing the receptor availability level. We then performed data reduction and CBDA to power rank the predictive brain voxels.

**Results:** CBDA classified migraineurs from HC with accuracy, sensitivity, and specificity above 90% for whole-brain and region-of-interest (ROI) analyses. The most predictive ROIs for μOR were the insula (anterior), thalamus (pulvinar, medial-dorsal, and ventral lateral/posterior nuclei), and the putamen. The latter, putamen (anterior), was also the most predictive for migraine regarding DOR D2/D3 BP_ND_ levels.

**Discussion:** CBDA of endogenous *μ*-opioid and D2/D3 dopamine dysfunctions in the brain can accurately identify a migraine patient based on their receptor availability across key sensory, motor, and motivational processing regions. Our ML-based findings in the migraineur’s brain neurotransmission partly explain the severe impact of migraine suffering and associated neuropsychiatric comorbidities.

## Introduction

Migraine is a pain disorder with a prevalence of more than one billion people worldwide (Global Burden of Disease GBD, 2017). It dramatically impacts the patients’ daily lives with frequent headache attacks, neuropsychiatric comorbidities, and the potential for substance abuse when unremitted, especially opiates (Bigal et al., 2009; Buse et al., 2012; Adams et al., 2015; Lipton et al., 2020). Because of this significant sensory and cognitive-motivational dysregulation in migraine, the endogenous *μ*-opioid and D2/D3 dopamine (DA) molecular mechanisms have recently been investigated as the potential main culprits of the disorder (Zubieta et al., 2002; De Felice et al., 2013; Martikainen et al., 2013; Jassar et al., 2019). Pharmacologically, they are the targets for the action of the most potent exogenous analgesic and psychotic drugs available. Positron emission tomography (PET) studies with [^11^C]Carfentanil, a selective *μ*-opioid receptor (μOR) agonist radiotracer, have demonstrated *in vivo* a decrease in μOR availability (non-displaceable binding potential [BP_ND_], where BP_ND_ is equal to the distribution volume ratio, DVR, minus one; BP_ND_ = DVR-1) in the brains of episodic migraineurs during headache attacks and allodynia (DaSilva et al., 2014a; Nascimento et al., 2014a). The μOR has a high affinity for the *μ*-opioid peptides enkephalins and beta-endorphin. The measure of specific uptake of [^11^C]Carfentanil, BP_ND_, decreases when there is an increase in endogenous *μ*-opioid (peptides) release and *vice versa*. The migraine attacks were also accompanied by an increase in DA D2/D3 receptor (DOR) BP_ND_ measured by [^11^C]Raclopride in the basal ganglia; and the longer the history and recurrence of migraine attacks, the lower the ictal (i.e., during the migraine attacks) endogenous DA release (DaSilva et al., 2017). Interestingly, migraine has been associated with a higher prevalence of DA-deficient disorders, including Restless Legs Syndrome and Parkinson’s disease (PD) (Cervenka et al., 2006; Scher et al., 2014).

A growing number of laboratories are attempting to translate migraine and chronic pain neuroimaging data to a more precision-oriented clinical approach by objectively classifying patients via potential brain biomarkers (Zhang et al., 2016; Lamichhane et al., 2021; Dumkrieger et al., 2022; Hsiao et al., 2022; Wang et al., 2022). Machine learning (ML) methods have been firstly implemented in migraine research with electroencephalography (EEG) (Frid et al., 2020) and structural/functional magnetic resonance imaging (MRI) (Chong et al., 2017; Ferroni et al., 2020; Hong et al., 2020). However, due to its cost and protocol complexity, molecular pain studies with PET have been scarce and limited to ML approaches specific to a single region of interest or a single radiotracer (Torrado-Carvajal et al., 2021). A multimodal ML technique would be of immense clinical value to characterize migraine based on its broad neurotransmitters-receptor interactions and multiple brain regions associated with it.

**FIGURE 1:**
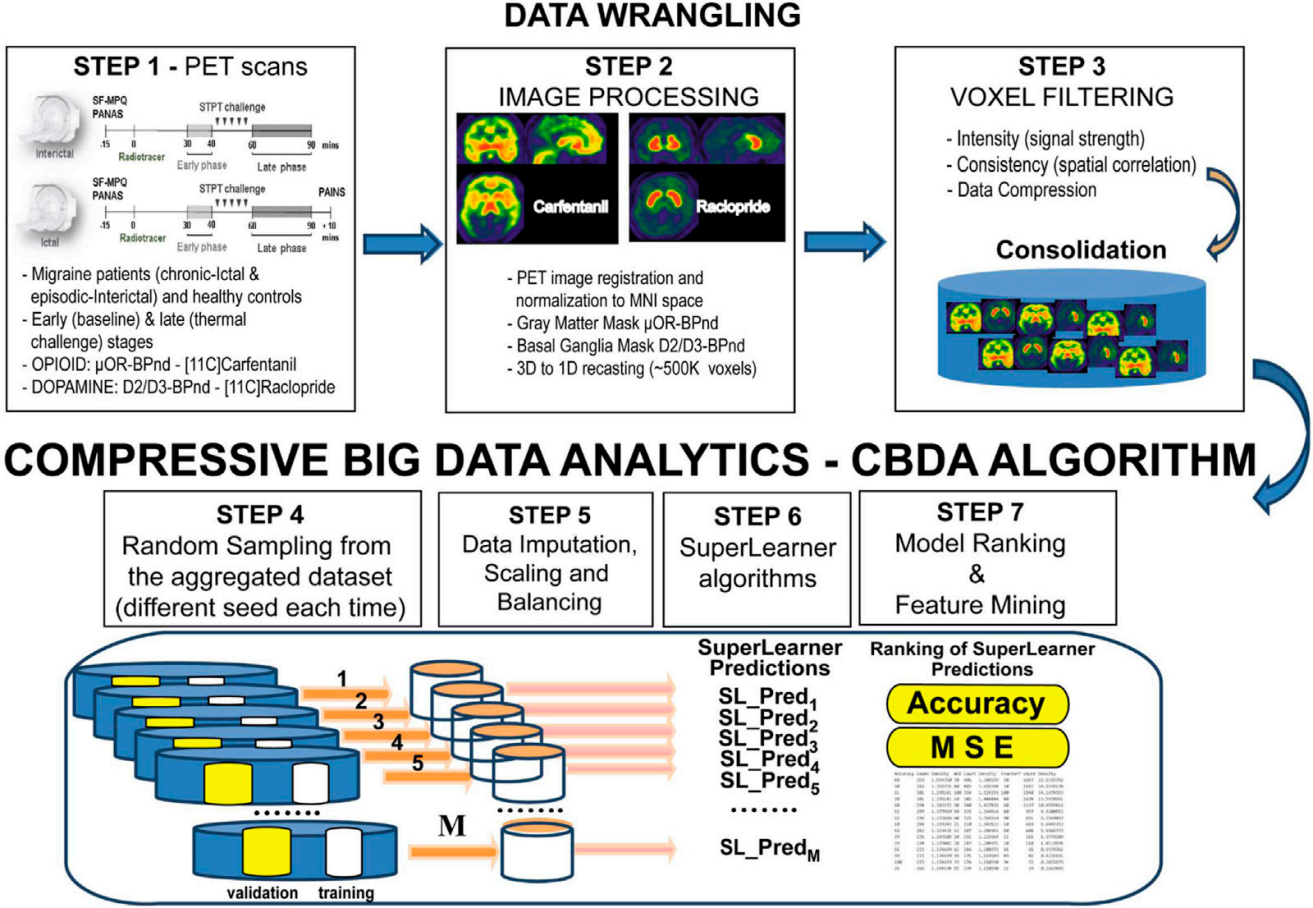
End-to-end representation of the CBDA protocol. The initial data wrangling (Steps 1-3) is data-dependent, while the remaining steps (Steps 4-7) of the CBDA protocol are data-independent

This novel study has been conducted following best practices in algorithm development in nuclear medicine and artificial intelligence (AI) (Bradshaw et al., 2021). We use a promising semi-supervised machine learning (ML) technique, called Compressive Big Data Analytics (CBDA) (Marino et al., 2018; Marino et al., 2020), to identify predictive migraine biomarkers spatially at the molecular level *in vivo*. The CBDA protocol exploits the concepts of subsampling and ensemble predictors to investigate the core principles of distribution-free and model-agnostic methods for scientific inference based on Big Data sets. Ensemble predictor algorithms and subsampling or bootstrapping use common approaches for objective function optimization, quantification of noise, bias, prediction error, and variance estimation during the learning process. CBDA combines in a unique way function optimization and statistical inference. An end-to-end representation of the Migraine PET CBDA protocol is shown in Figure 1. The initial data wrangling (Steps 1-3, Figure 1) is data-dependent, while the remaining steps (Steps 4-7, Figure 1) of the CBDA protocol are data-independent. The ensemble predictor embedded in CBDA is SuperLearner (van der Laan et al., 2007), a data-adaptive black-box ML approach to prediction and density estimation. SuperLearner uses cross-validation to estimate the performance of multiple ML models. Currently, the SuperLearner library comprises 60 different classification, regression, and artificial intelligence (AI) algorithms to strengthen its predictive power and sensitivity (see *Material and Methods* section for details on the Migraine CBDA workflow).

A CBDA R package (Marino and Dinov, 2018) can be deployed on a desktop/laptop environment and a high-performance computing platform using the LONI graphical pipeline environment (Rex et al., 2003). Our previous studies (Marino et al., 2018; Marino et al., 2020) showcased the robustness, efficiency, accuracy, and viability of our first-generation CBDA protocol on small-medium-large size data.

This study enhances the protocol to handle PET imaging data specific to migraine and brain double neurotransmission-receptor data. We first perform extensive data wrangling on the 3D imaging data and recast them into 1D vectors of PET intensities. A thresholding protocol has been designed and implemented to reduce the large 1D vector of PET intensities (see *Material and Methods* section for details).

Across the different predictive analytics tasks, CBDA yielded accurate results with robust sensitivity and high specificity metrics. The identified top predictive brain regions of interest (ROIs) confirmed existing results in the migraine literature (DaSilva et al., 2014b; DaSilva et al., 2017; Jassar et al., 2019; Kim et al., 2021). These results suggest new potential avenues for dissecting the molecular brain mechanisms underlying migraine and its treatment. PET imaging and the open-source classifier CBDA point to the importance of using these techniques in synergy for accurate mining *in vivo* of the main biomarkers of migraine regarding endogenous *μ*-opioid and dopamine D2/D3 transmitter-receptor dynamics in the migraine brain.

## Materials and methods

### PET data collection protocol

Thirty-eight migraine subjects, seven chronic migraine (CM), 31 episodic migraine (EM), and 23 healthy control (HC) participants, 20–64 years old, underwent structured clinical phone screening for formal diagnosis as per the International Headache Society Classification (ICHD-3-beta) (Headache Classification. Committee of the International Headache Society, 2004), were studied (see Table 1 for details). The clinical samples (7 chronic migraine, eight episodic migraine and eight healthy controls) used in this study overlaps with the sample utilized in our previous publications (DaSilva et al., 2014a; DaSilva et al., 2017; Jassar et al., 2019). All participants were recruited through local advertisement at the University of Michigan, Internet and flyers on bulletin boards. The exclusion criteria included pregnancy, opioid or hormonal contraceptive use 6 months prior to recruitment, other chronic pain disorders, as well as major systemic medical or psychotic disorders. Additionally, all subjects underwent a urine drug screening to ensure the absence of substance abuse, including cocaine, amphetamine, methamphetamine, marijuana, and opioids. More details on inclusion and inclusion criteria please refer to previous publications (DaSilva et al., 2014a; DaSilva et al., 2017; Jassar et al., 2019).

**TABLE 1:** Data and CBDA specifications.

			Carfentanil CFN opioid subjects			Raclopride RCL dopamine subjects		
Healthy			23 (Female: 17, Male: 6)			10 (Female: 5, Male: 5)		
Migraine			38 (Female: 23 [EM^(*)^], 6 [CM^(*)^], Male: 8 [EM], 1 [CM])			12 (Female: 7 [EM]		
						Male: 5 [EM])		
Images (Early and Late Stages)			138^(^**^)^			60^(^**^)^		

	Whole Brain	ROI					Whole Brain?	ROI
	Gray Matter mask [voxels]	Insula [voxels]		Thalamus [voxels]	Putamen [voxels]		Basal Ganglia mask [voxels]	Putamen [voxels]
^(^***^)^Unfiltered data	510,340	5,318		2,320	2,402		510,340	2,402
Threshold data	4,675	1,926		1,864	1,547		2,295	2,101
Threshold value	2	1.1		1.1	1.1		2	1.1

^(^*^)^CM: chronic migraine, EM: episodic migraine^(^**^)^8episodic migraineurs underwent both CFN, and RCL, scans during interictal and ictal states. Thus, the images are actually referring to 122 and 44 unique subjects, respectively for CFN, and RCL, scans.^(^***^)^The first row here represents the complete # of voxels for each “region” (i.e., WB, insula, Thalamus, Putamen), the second row is the # of voxels left for predictive analytics if we use the threshold in the third row.

A total of 198 PET images obtained after scanning 61 subjects, comprising two cohorts of 38 migraines subjects (mean 28.84 ± standard deviation 8.4; nine men and 29 women) and 23 HCs (mean 26.27 ± standard deviation 6.25, six men and 17 women) during both rest and cutaneous thermal pain threshold were used to train and validate the CBDA predictive model.

All 61 subjects were scanned outside (interictal) and/or during (ictal) migraine attacks using PET with the selective *μ*-opioid receptor (μOR) radiotracer [^11^C]Carfentanil. Additionally, a subset of 22 subjects were scanned using PET with the selective dopamine D2/D3 receptor (DA) radiotracer [^11^C]Raclopride. Specifically, seven chronic migraineurs underwent ictal CFN PET (; a total of 14 images). Thirty-one episodic migraineurs underwent interictal CFN PET (62 images total). Twelve out of 31 episodic migraineurs had interictal RCL PET (24 images total) and eight of 31 did ictal CFN/RCL PET (32 images total). In total, there were 132 images for all migraineurs.8 episodic migraineurs underwent both CFN and RCL scans during interictal and ictal states. Thus, the images are actually referring to 122 and 44 unique subjects, respectively for CFN and RCL scans.

### PET acquisition protocol

PET scans with a radiotracer [^11^C]Carfentanil, a selective *μ*-opioid receptor radiotracer, or [^11^C]Raclopride, a selective radiotracer for DA D2/3Rs, were acquired with a Siemens HR + scanner (Knoxville, TN) in 3-D mode with septa retracted (DaSilva et al., 2014a; Nascimento et al., 2014b; DaSilva et al., 2017; Jassar et al., 2019). Each image was reconstructed using the full-width at half maximum (FWHM) resolution of ∼5.5 mm in-plane and 5.0 in the *z*-axis. The total dose injected of [^11^C]Carfentanil was 15 mCi (555 MBq), with a maximum mass of 0.03 μg per kilogram of body weight. Each [^11^C]Raclopride dose of 15 ± 1 mCi (555 ± 37 MBq), ≤50 μg, was administered. 50% of this dose was administered as a bolus, followed by a continuous and constant infusion of the remainder to quickly achieve the steady-state tracer levels. Twenty-one PET images were acquired over 90 min while increasing duration (30 s up to 10 min). The PET scan with [^11^C]Carfentanil included a resting early-phase (5–40 min) without stimulation, followed by a sustained thermal pain threshold (STPT) challenge for the late phase (45–90 min) (thermal pain threshold response). Regarding the PET with [^11^C]Raclopride, participants were resting without challenge during the early phase (30–40 min), followed by the STPT challenge for the late phase (60–90 min). During the STPT challenge, heat stimuli were delivered to the forehead area ipsilateral to the headache using a 16 mm^2^ thermal probe (Pathway model; MEDOC, Ramat Yishai, Israel). The temperature was increased from a baseline of 32°C (increasing 1°C/s). Participants were instructed to click a mouse at the first perception of pain to instantly return temperature to baseline level. The heat stimuli recurred every 10 s for 20 min (40–60 min post-radiotracer administration).

The highest temperature allowed by the device during the challenge was 50°C. The time between scans, assuming the use of CFN to RCL, could be up to 10 min to ensure the tracers were optimally delivered. The STPT was applied to the trigeminal nerve region, which is the most common clinical pain location and allodynia in migraineurs. The patients’ heads were firmly stabilized with a soft self-adherent compression bandage wrap and headrest before each scan. Nonetheless, images also underwent attenuation correction and quality control before registration. The probe used for the STPT was attached to the headrest via a plastic and sturdy holder adjusted to comfortably rest on the patients’ faces. These are the average molar activities for the data collected across the different studies: i) CFN (2011–2014) — 28.57Ci/μmol [1,057 GBq/µmol], ii) Rac (2011–2014) — 20.75Ci/μmol [767.75 GBq/µmol], iii) CFN (2017-2020) — 117.12Ci/μmol [4333.44 GBq/µmol].

### PET data preprocessing

PET images were reconstructed using interactive algorithms into a 128 * 128 pixel-matrix in a 24 cm diameter field of view with attenuation and scatter corrections. A patient motion was corrected by performing a linear co-registration of the frames of dynamic PET images. For each participant, PET images were transformed into two sets of parametric maps: 1) K_1_, a tracer transport measure usually used for PET-MRI image co-registration and normalization, and 2) BP_ND_ (non-displaceable binding potential), a receptor-related measure estimated by applying the Logan plot (Logan et al., 1996). Both BP_ND_ and K_1_ images were then transformed to the Montreal Neurological Institute (MNI) standard space using the DARTEL tool in SPM8 (Ashburner, 2007). The normalized BP_ND_ images were resampled to 2-mm voxels and smoothed with a Gaussian kernel (3 * 3 * 2 mm). The Logan plot output is distribution volume ratio (DVR), where here the ratio means relative to a reference region, which in our case is the occipital cortex for Carfentanil and cerebellar gray matter for Raclopride, The reference region value is always 1.0. Thus, the normalized BP_ND_ values used by CBDA for predictive analytics are calculated subtracting one to DVR (i.e., BP_ND_ = DVR - 1).

### PET data wrangling

After the PET data have been acquired, we developed and implemented the following protocol for our PET data analysis and predictive analytics. We first recast each 3D brain scan into a 1D array of 510,340 voxels and eliminate voxels with DVR (BP_ND_ +1) < 1.1. Two masks have been used, an CFN mask for CFN data (Grey Matter mask) and an RCL mask for RCL data (Basal Ganglia mask). These masks were generated using the Automated Anatomical Labeling (AAL) brain atlas (Tzourio-Mazoyer et al., 2002). Both masks are applied in MNI space. These masks include only the brain tissue where each of the radiotracers most prominently binds to the appropriate neuro-receptors (Baumgartner et al., 2006; Karjalainen et al., 2017). These different masks set voxel intensities outside the ROIs to zero, leaving the 1D array size unchanged. Therefore, CFN and RCL masks are specific to the gray matter and basal ganglia, respectively. Regarding the ROI analysis, ROIs from AAL atlas have been used for *Thalamus*, *Putamen*, and *Insula*. These ROIs are applied in MNI space. We did further analysis for *Thalamus* by mapping each predictive voxel coordinate in MNI space to *Talairach* space to look up the sub-nuclei (Lancaster et al., 2000). Before the data can be used for predictive analytics, we used each PET image data across the different phases (early and late) by thresholding each voxel intensity across the cohorts of subjects using *ad hoc* masks (i.e., Grey Matter and Basal Ganglia) and a newly designed thresholding function (see Table 1 and next section).

### *The* BP_ND_ *thresholding function*

BP_ND_ intensity thresholding is based on two parameters: threshold and consistency. The thresholding function returns a reduced 1D array based on:

- *consistency* (*%*)*:* given a set of subjects, we want the pixel location to be above the
- *intensity threshold* for at least that % of subjects

We apply the *thresholding* function to each set of images from the two experiments, combining the early and late phases. The details on the thresholding protocol and on the datasets used for predictive analytics are given in Table 1. The consistency value used in the thresholding function is 80%. The threshold for the whole brain analysis was set to 2.0, while we used a less stringent threshold for the ROI analyses (i.e., 1.1) since each ROI has a lot less voxels and a 2.0 threshold would eliminate most of the BP_ND_ values from the analysis. We then perform our CBDA protocol on the reduced PET data.

### PET data predictive analytics and CBDA implementation

The study design for our CBDA protocol on the PET imaging data comprised multiple experiments to be performed on each dataset. Details on the CBDA protocol are available in our previous publications (Marino et al., 2018; Marino et al., 2020) and Figure 1. Here we highlight the main steps and specifications implemented for analyzing the PET imaging data after data wrangling has been performed (as described in the previous section, in Figure 1 and in Table 1).

The CBDA subsampling module samples and returns subsets of cases/rows and features/columns used to build the smaller training sets. If needed, imputation (Stekhoven and Buhlmann, 2012), normalization (Becker et al., 1988) and balancing (Chawla et al., 2002) are performed on each sample. In this study, there was no need to perform any of these adjustments since each set of voxels with their binding potential values has no missingness, and the data are already normalized. The voxels are then paired to the binary outcome vector for each patient’s PET image (i.e., migraine = 1 vs healthy = 0). The CBDA settings for the subsampling module for all the experiments are the following:

i) M (number of samples from the big dataset) = 5000,
ii) We performed internal cross-validation; thus the Case Sampling Rate-CSR (i.e., fraction of cases/rows to sample from the original training/learning set) is set to 138 for the Opioid experiment and to 60 for the Dopamine experiment,
iii) Feature Sampling Rate—FSR (i.e., a fraction of features/columns to sample from the original training/learning set) is set to 20.

Each of the 5,000 jobs comprises a training sample the size specified in the CSR and FSR. Due to the small number of images/patients, only internal cross-validation is performed.

The sampled pairs are then passed and analyzed by our ensemble predictor (i.e., the *SuperLearner*-SL, see (Van Der Laan and Rubin, 2006; van der Laan et al., 2007; Polley et al., 2017) for details) that combines many different pre-defined algorithms into a single predictive model. The *SuperLearner* algorithm has a large library of classification algorithms that we use to generate our predictions (e.g., Generalized Linear Model (McCullagh and Nelder, 1989), General Additive Model (Hastie and Tibshirani, 1990), Ridge and Lasso Regression (Friedman et al., 2009; Friedman et al., 2016), Random Forest (Breiman, 2001), Support Vector Machine (Hearst et al., 1998), Bayes Auto Regressive Tree (Chipman et al., 2010; Kapelner and BartMachine, 2013)). The SuperLearner ensemble predictor uses a Non-Negative Least Squares loss function to build a weighted linear combination of each algorithm selected to generate the best ensemble prediction (van der Laan et al., 2007). Cross-validation is always performed by default for each algorithm and for the ensemble predictor. We use the default 1 to 10 ratio. A performance metric (accuracy) is then used to rank each prediction. After ranking, we select for features with high frequency (signal) among the top-ranked predictive models and across the M subsamples. The outcome of the CBDA is a set of key features/voxels for prediction/classification, which we then test in a set of nested SuperLearner models for overfitting. We use the top 50 predictive voxels for each CBDA experiment. The current CBDA R package (Marino and Dinov, 2018) completes each instance/job within 2–7 min. Our CBDA technology becomes feasible and scalable (e.g., thousands of instances/jobs can be run within 1–2 h) by combining *short-burst* completion times for each instance/job with the access to free large scale computational resources through multicore servers, such as the LONI pipeline environment (Dinov et al., 2014).

### Features ranking

**FIGURE 2:**
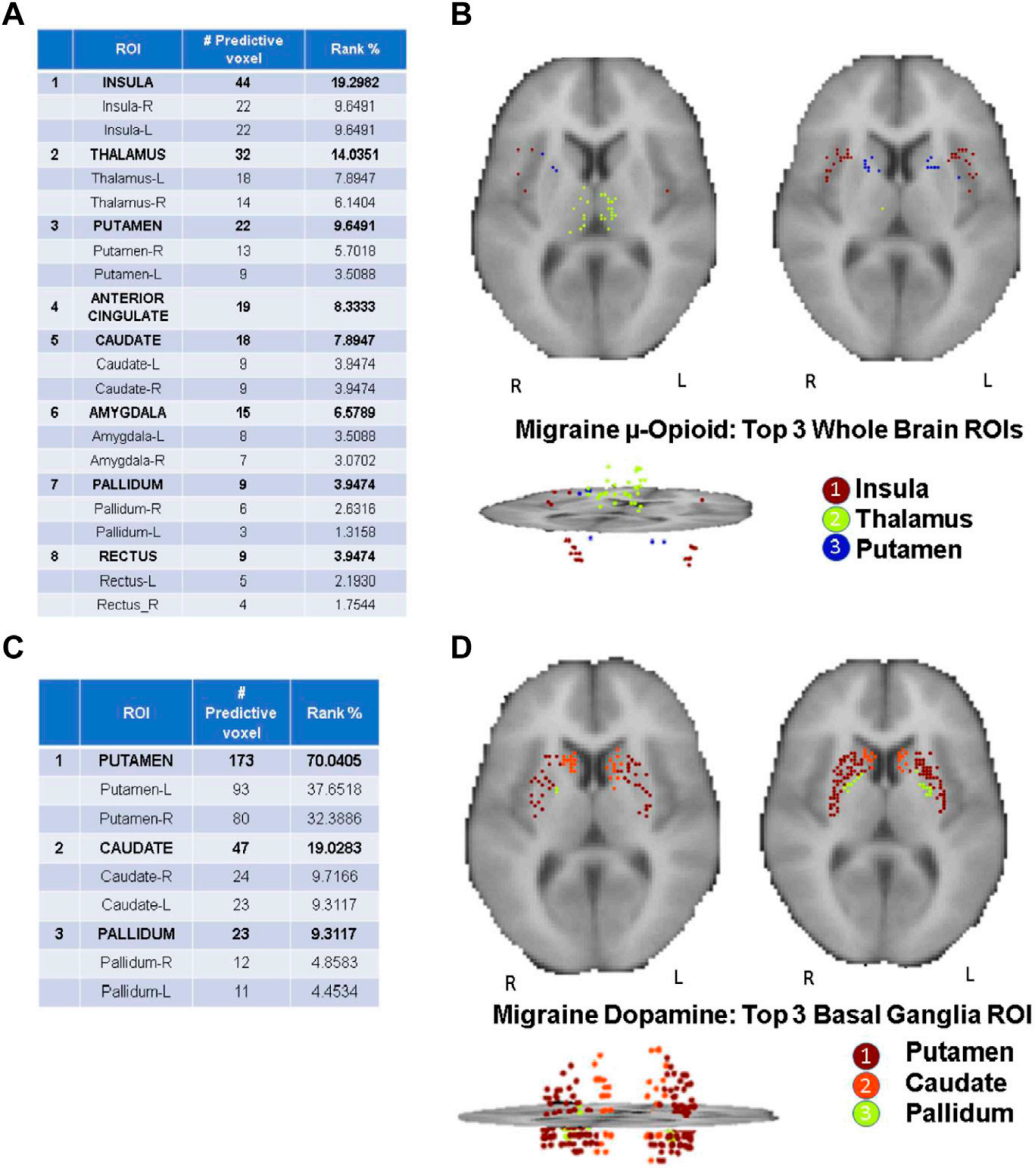
CBDA results for the Whole Brain analysis. The binding potentials of the top 50 predictive voxels across 5 replications from the top 3 ROIs are grouped together by their respective ROI descriptions (tables in the figure). Each voxel is then plotted in the 2D rendering of the brain (see the images above the tables). The ranking in the tables is based solely on the fraction of the voxels belonging to their respective ROI. Higher % can be interpreted as a ROI with more predictive power. Panel **(A, B)**: µ-Opioid (Carfentanil) results, based on the Grey Matter mask. Panel **(C, D)**: Dopamine D2/D3 (Raclopride) results based on the Basal Ganglia mask. *Note*: the search for the predictive coordinates for Thalamus and sub-nuclei was performed in Talairach space.

**FIGURE 3:**
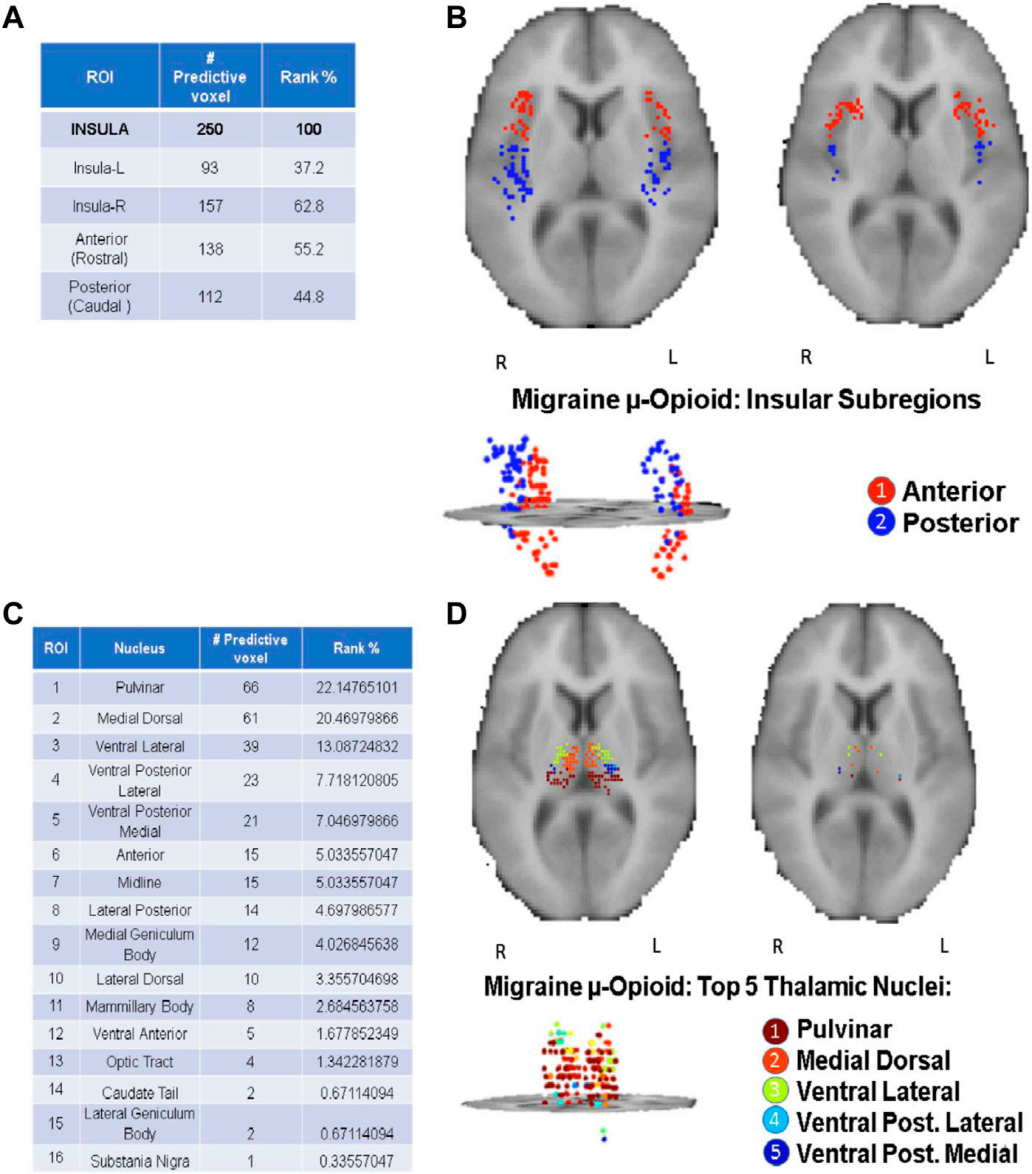
CBDA results for the Insula and Thalamus ROI. The binding potentials of the top 50 predictive voxels across 5 replications from the Insula [Panels **(A, B)**] and Thalamus [Panels **(C, D)**]. The binding potentials of the top 50 predictive voxels across 5 replications are grouped together by their respective sub-regions or nuclei, wherever possible (tables in the figure). Each voxel is then plotted in the 2D rendering of the ROI (see the images above the tables). The ranking in the tables is based solely on the fraction of the voxels belonging to their respective subregions/nuclei. Higher % can be interpreted as a subregion/nucleus with more predictive power. Panels **(A, B)**: µ-Opioid (Carfentanil) results for Insula. Panel **(C, D)**: µ-Opioid (Carfentanil) results for Thalamus. *Note*: the search for the predictive coordinates for Thalamus and sub-nuclei was performed in Talairach space.

**FIGURE 4:**
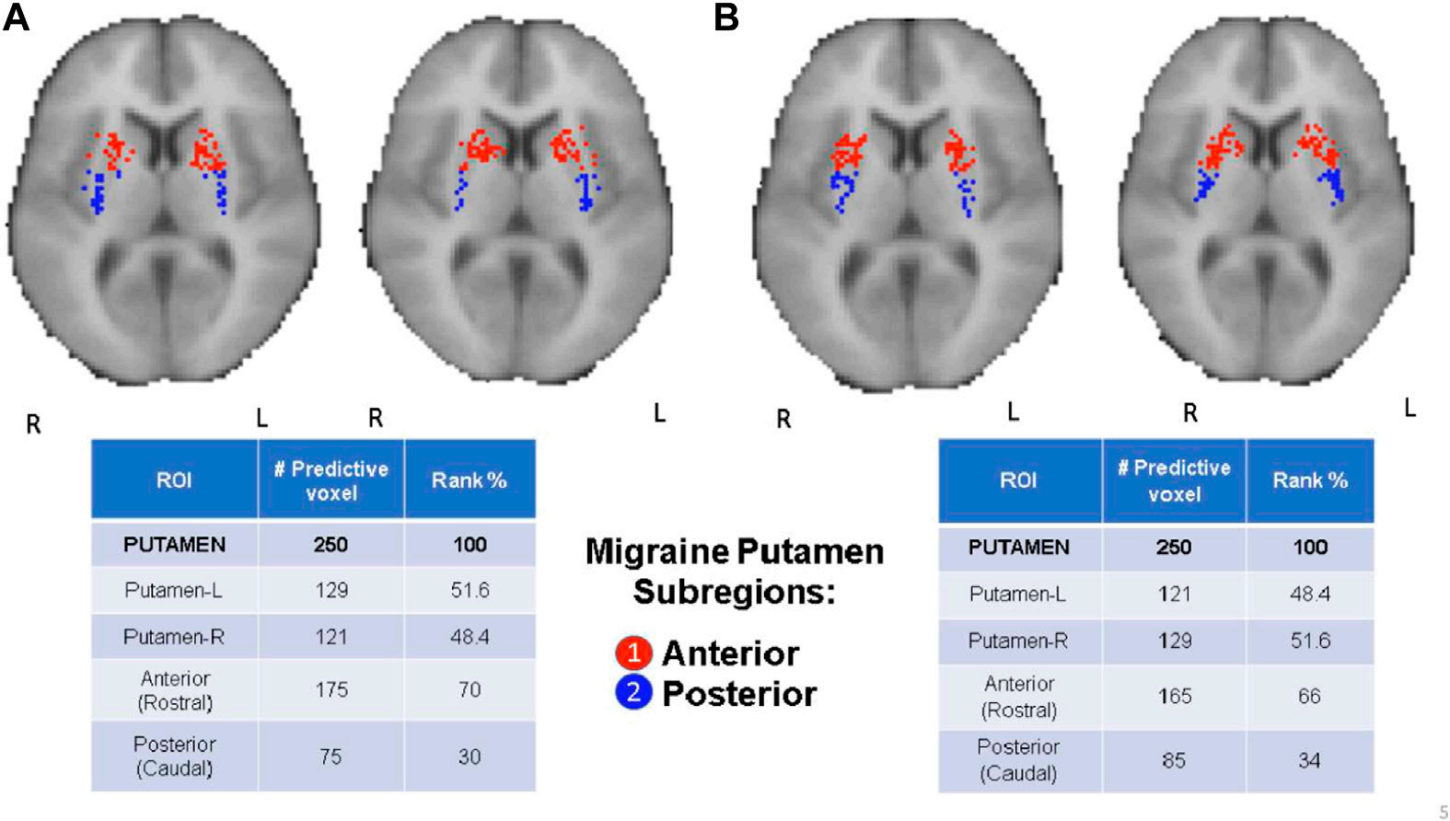
CBDA results for the Putamen ROI. The binding potentials of the top 50 predictive voxels across 5 replications are grouped together by their respective sub-regions or nuclei, wherever possible (tables in the figure). Each voxel is then plotted in the 2D rendering of the ROI (see the images above the tables). The ranking in the tables is based solely on the fraction of the voxels belonging to their respective subregions/nuclei. Higher % can be interpreted as a subregion/nucleus with more predictive power. Panel **(A)**: µ-Opioid (Carfentanil) results. Panel **(B)**: Dopamine D2/D3 (Raclopride) results.

As highlighted in the Results section, the CBDA protocol has been performed 5 times on each dataset to ensure robustness of the predictive voxels selected. Once a filtered dataset is passed to the CBDA protocol, each time the top 50 predictive voxels from each replication are merged together in a set of 250 non-unique voxels. There is always a 30% up to 40% overlap across the 250 top predictive voxels across the five replications. Usually the maximum accuracy is achieved with less than 50 voxels for each replication, but we list 50 for completeness. We then label each voxel selected based on their ROI (see Figure 2), sub-region or nucleus (see Figures 3, 4), depending on the data used. Each table then rank the voxels by the frequency of occurrence of their respective labels (e.g., ROI, sub-regions, and nuclei). A total of 30 CBDA experiments have been performed, each one generating 5,000 subsamples.

## Results

The CBDA results highlight top predictive voxels grouped by region-of-interest (ROI) of non-displaceable binding potential (BP_ND_) for 198 PET images from migraineurs and healthy controls (HC) (2/1 ratio) during rest and thermal pain challenge. Subjects were scanned with the selective μOR radiotracer [^11^C]Carfentanil and the selective DOR D2/D3 radiotracer [^11^C]Raclopride. The first sets of top predictive voxels for μOR and D2/D3 BP_ND_ were returned after performing CBDA on the whole brain masks filtered datasets (Figure 2). Based on the results described in Figure 2, the voxels of each top predictive ROI are isolated, and CBDA is independently performed on each ROI to identify predictive sub-regions (namely nuclei, see Figures 3, 4). To ensure robustness, the CBDA protocol has been performed five times on each dataset, and the top 50 predictive voxels from each replication have been merged (see *Feature Ranking* in the Methods section for details). There is significant overlap on the top predictive voxels across the five replications. The tables embedded in Figures 2–4 recapitulate most of the 250 top predictive voxels ranked by the frequency of occurrence and grouped by ROIs. The accuracy returned by the internal cross-validation over the set of experiments varied between 80% and 95%, with sensitivity and specificity within 73% and 99% (see Table 2 for detailed results on the Whole Brain Opioid data and Supplementary Text S1 for details on everything else). The following subsections describe the results in detail.

**TABLE 2:** Summary of the CBDA results on the five replications performed on the whole brain for the Carfentanil data (Opioid Grey Matter Mask).

Replication 1	Replication 2	Replication 3	Replication 4	Replication 5
Accuracy: 0.9058	Accuracy: 0.9348	Accuracy: 0.9783	Accuracy: 0.9058	Accuracy: 0.9638
95% CI: (0.8443, 0.9489)	95% CI: (0.8798, 0.9697)	95% CI: (0.9378, 0.9955)	95% CI: (0.9603, 0.9998)	95% CI: (0.9175, 0.9881)
Sensitivity: 0.7391	Sensitivity: 0.8043	Sensitivity: 0.9348	Sensitivity: 0.9783	Sensitivity: 0.8913
Specificity: 0.9891	Specificity: 1	Specificity: 1	Specificity: 1	Specificity: 1

### Top predictive ROIs: opioid vs. dopamine whole brain analysis

Figure 2 Illustrates the detailed results returned by CBDA using the Gray Matter and Basal Ganglia masks on the PET images of the entire brain. Figures 2A,C list the top predictive voxels grouped by their respective ROIs and the frequency of each voxel selected as a top predictive one across the five different replications. Due to the binding profile of the Carfentanil tracer (i.e., more diffused receptors in the brain), the *μ*-opioid CBDA results are scattered over more ROIs than the Raclopride analysis which mainly binds in the basal ganglia. Figure 2A table lists the top eight predictive ROIs in the *μ*-opioid analysis, representing ∼73% of the top predictive voxels.

The top three ROIs selected are *Insula*, *Thalamus,* and *Putamen*. If we compare these results with the DOR D2/D3 analysis results (shown in Figure 2C table), *putamen* represents alone almost the same percentage of predictive voxels (i.e., ∼70%) compared to the top eight ROIs in the *μ*-opioid analysis. The binding potential levels of the top three predictive ROIs returned by the Dopamine CBDA analysis were able to predict migraine patients from healthy controls with almost 100% accuracy. The detailed results for the *μ*-opioid analysis are shown in **Supplementary Text S1 (Panel A).** The min-max of the 95% confidence intervals for the accuracy across the five replications are 84% and 99%. Similarly, the min-max values are 0.74 and 0.97 for sensitivity and 0.98 and one for specificity, with positive and negative predicted value rates approaching one in most replications. Although we list and merge the top 50 predictive voxels across the five replications (Figure 2A), the best results can be achieved using as low as the top seven (replication two) up to the top 32 voxels (replication four).

The detailed results for the Dopamine analysis are shown in **Supplementary Text S1 (Panel B).** The min-max of the 95% confidence intervals for the accuracy are 94% and 100%, with 100% sensitivity and specificity. The table in Figure 2C shows the top predictive voxels across the five replications grouped by sub-regions. The best results shown in Figures 2C,D can be achieved using as low as the top three (replication one, two, and five) and up to the top 17 voxels (replication 2).

Figures 2B,D portray the 2D and 3D spatial distributions of the voxels of the top three predictive ROIs for the Opioid and Dopamine analyses, respectively.

### Top predictive nuclei: opioid vs. dopamine ROI analysis

CBDA analyses based on the Whole Brain and Basal Ganglia generated separate predictive analytics independently for each of the top predictive ROIs, as illustrated in Figure 2. We focused on the top three ROIs for the *μ*-Opioid experiment (i.e., Insula, Thalamus, and Putamen), and only at the Putamen ROI for the Dopamine experiment due to its preeminence in the initial basal ganglia CBDA analysis.

Figure 3 shows the CBDA results on *Insula* and *Thalamus* for the Opioid experiment, while Figure 4 compares the *Putamen* region between the two experiments. The top predictive voxels were then mapped to ROI sub-regions or nuclei.

Detailed results for the Opioid Insular sub-regions, Thalamus nuclei, and Putamen sub-regions analyses (see **Supplementary Text S1**—**Panel C, D, E, and F)** have similar min-max of the 95% confidence intervals for the accuracy within 97% and 100%, with 100% sensitivity and specificity. The tables in Figures 3, 4 show the top predictive voxels across the five replications grouped by sub-regions and nuclei. The best results shown in the tables can be achieved using as low as the top three up to the top 11 voxel, across the five replications.

Figures 3B,D, and the top row of Figures 4A,B portray the 2D and 3D spatial distributions of the top predictive voxels of the ROIs analyzed, namely Insula, Thalamus, and Putamen for the three *μ*-opioid data and only putamen for the Dopamine D2/D3 data, respectively.

## External cross validation results

Due to the limited number of subjects, the results showcased so far in the study are based on internal cross validation analyses. As a proof of concept, **Supplementary Text S3** showcases a tentative CBDA external cross validation experiment performed on the Grey Matter mask for the whole brain BP_ND_ *μ*-opioid Carfentanil data. Due to the low number of subjects, it was not feasible to obtain a large enough and balanced subsample for the Dopamine BP_ND_ dataset. In order to achieve meaningful results, we used a 20%–80% split between validation and training set. Based on the demographics (23 healthy, 38 migraine) and since each subject has two PET images (early and late), we selected four healthy (2 males, two females, eight PET images [early/late]) and eight migraine (4 males, four females, 16 PET images [early/late]) subjects. We then performed five CBDA replications on the Grey Matter mask data (Whole Brain), using the trained model to predict the outcomes (0 = healthy, 1-migraine) of the external validation set. The CBDA external cross validation results confirmed our main results (see **Supplementary Text S3** for details).

### Predictive analytics results for interictal only patients images

As a proof of concept, similarly to the external cross validation effort, we perform CBDA on the only [11C]Carfentanil BP_ND_ *μ*-opioid data of episodic migraineurs during the interictal state. Individuals with migraineurs experience various types of discomfort, including sensory hypersensitivities and emotional/cognitive dysfunction, even during headache-free days (Vincent et al., 2022). Previous neuroimaging studies have demonstrated some level of neural abnormalities associated with interictal sensory alterations, susceptibility to migraine attack and severity (Ashina et al., 2021). Thus, we analyzed the data by only including interictal patients/images, which will provide a better insight into the molecular substrate of migraine burden and help guide treatment strategy. Based on Table 1, we included 108 images from 31 patients and 23 HC for the CBDA protocol.

The CBDA results for the interictal patients/images confirmed our main results (see **Supplementary Text S4** for details).

## Discussion and conclusions

This work is the result of an interdisciplinary effort across different domain experts. In order to avoid the common pitfalls emerging in AI studies, we focused on reproducibility and transparency. In an effort to promote reproducibility and transparency, we make our code implementations open source and available on public repositories (Marino and Dinov, 2018; Marino and Dinov, 2019). We fully disclose our datasets characteristics and limitations as well as the potential challenges regarding spatial correlation and sample sizes.

There are several limitations when predictive analytics techniques are applied to very high resolution data, such voxel intensities in PET scan measures (like in this study). There is an intrinsic spatial correlation that can be partially eliminated by any data wrangling performed after data has been collected. Of course, this limitation can also be alleviated by a significant progress in PET scan technology. In this study, we do not cluster the voxels before any predictive analytics is performed (like most of the methods in the current literature, see (Peng et al., 2014; Retico et al., 2015; Mete et al., 2016; Klyuzhin et al., 2018) for examples). We apply our CBDA method to the single voxel intensities (upon some thresholding), and then guide the post-optimization clustering by the ROI classification, as well as MNI mapping of the sub-regions (whenever available). Our approach does not bias the predictive analytics by any *a priori* distance-based clustering approaches. We retain the original voxels and ROI coordinates to facilitate any clinical translation of the results.

Another limitation is specific to the data collected in this study, rather than to the methodology implemented here. There is a limited supply of external independent validation datasets due to the very recent use of Carfentanil and Raclopride tracers in PET migraine studies. This scarcity may present some obstacles when it comes to obtaining precise and dependable findings to compare with the present results. However, it is hopeful that future research will broaden the usage of these tracers and offer more data for the purpose of validation.

As a final limitation of this study, we want to stress that results obtained on the Whole Brain Dopamine data (Basal Ganglia Mask), as well as on all the individual ROI predictive analytics, are very similar in terms of accuracy, sensitivity and specificity, likely due to the small number of co-localized voxels which drive the correct classification. We are gathering more subjects to independently validate these results on a larger cohort with external validation settings.

The analysis using the CBDA protocol of our large PET dataset accurately identified migraine patients’ brains from those of healthy controls based on their *μ*-opioid and dopamine D2/D3 receptor availability measure (BP_ND_). The accuracy, sensitivity, and specificity were above 90% for both whole-brain and region-of-interest (ROI) analyses. The most predictive ROIs for *μ*-opioid were insula, Thalamus, and putamen. Putamen led the ranking for dopamine D2/D3, followed by caudate and *pallidum*, respectively. These are critical sensory, motor, and motivational processing regions that are also related to pain and migraine suffering.

The Compressive Big Data Analytics method used i) *subsampling and bootstrapping* for reducing the PET Big Data voxel space into smaller chunks, and ii) *ensemble predictors* for predictive analytics. Given the size of each PET image, we designed and implemented a *thresholding* function that provides significant data compression (up to 60 folds). This functionality improved the clinical predictive power and reduced the computational costs (e.g., CPU time, memory, and storage resources). This method augments the power of machine learning classification and artificial intelligence prediction using MRI and PET in pain and migraine (Chong et al., 2021; Torrado-Carvajal et al., 2021). In addition, the technique provides a robust approach for combining and evaluating heterogeneous data on *μ*-opioid and dopamine D2/D3 receptor availability (BP_ND_) in migraine.

The entire CBDA protocol was designed, implemented, and validated as a reproducible, open project using the statistical computing language R. The workflow ran on the LONI pipeline environment, a free platform for high-performance computing, which allowed the simultaneous submission of hundreds of independent components of the CBDA protocol. In addition, our results showcased the scalability, efficiency, and potential of CBDA to compress complex neuroimaging data into structural information leading to derived knowledge and translational action not only the basic diagnostic molecular profile but the ranking of potential treatment targets in the brain for migraine-related *μ*-opioid and dopamine D2/D3 dysfunction.

### Classification of migraine based on its central *μ*-opioid and dopamine D2/D3

PET studies have recently demonstrated that pain activates endogenous opioid and dopamine receptor-mediated neurotransmission in cortical and brainstem regions. The magnitude of the opioid and dopamine regional activations are related to the individual’s capacity to suppress sensory and affective elements of the acute pain experience (Zubieta et al., 2001; Scott et al., 2008), which can lead to maladaptations in the receptor availability in the same regions in chronic pain disorders (Martikainen et al., 2015). CBDA modeling of the data demonstrated a fingerprint of migraine in such pain’s *μ*-opioid and dopamine receptor network. For example, the insula is known to participate in pain perception and chronic pain (Mayr et al., 2021). It is divided into the anterior component, primarily related to limbic regions, including the amygdala, and plays a vital role in cognitive-emotional pain modulation. The posterior insula is associated with sensorimotor integration and receives nociceptive inputs from the Thalamus. This holistic role of the insular cortex in pain processing (Borsook et al., 2016) might explain its leading ranking in our ML classification model for migraine, the anterior insular slightly more than the posterior one. The dysfunctional activity in the insula is commonly linked to migraine, and its moment-to-moment variability of resting-state activity increases with structures on the ascending trigeminal somatosensory system in parallel with the severity of the attacks (Lim et al., 2021). There is also evidence of cortical neuroplasticity that correlates with the history and frequency of the attacks (Maleki et al., 2012; Woldeamanuel et al., 2019). At least in animal models, electric stimulation of the insula effectively reverses mechanical hypersensitivity, which can be abolished by pharmacologically blocking the *μ*-opioid receptors (MOR) system (Dimov et al., 2018). Such findings indicate the central role in the insular *μ*-opioidergic malfunction in migraineurs, and further studies can lead to the potential development of new migraine and pain therapies directly targeting the region (Bergeron et al., 2021; Liu et al., 2021).

Our second-ranking structure for *μ*-opioid dysfunction in migraine was the Thalamus. Thalamus is the relay structure in the brain that participates in multiple phases of migraine pathophysiology outside (interictal) and during (ictal) the attacks (Martinelli et al., 2021). Its highly specialized nuclei dynamically integrate with other regions in the brain associated with patients’ suffering. The leading thalamic nuclei in our study were the pulvinar and medial dorsal. They receive inputs from dura‐sensitive spinal trigeminal nucleus neurons and play a crucial role in the photophobia and allodynia during migraine (Hodkinson et al., 2016). The other implicated thalamic nuclei were the venteroposterior medial (VPM) and lateral (VPL) that are key in the processing of nociceptive inputs from cranial and extracranial structures during the attacks, respectively, and commonly reported in fMRI studies in migraine (Lim et al., 2021). In a recent study, chronic migraine patients scanned with PET during attacks under thermal challenge showed increased endogenous *μ*-opioid receptor-neurotransmission in the thalamic venteroposterior nuclei (Lim et al., 2021). Overall, the thalamic fingerprint of migraine confirms its involvement in the broad sensory dysfunction commonly noticed in migraineurs, including inefficient inhibitory pain modulatory responses and sensitization when exposed to multiple external stimuli such as thermal, mechanical, light, and sound (Lim et al., 2021). This broad *μ*-opioid receptor dysfunction in critical integrative thalamic nuclei explains the long list of symptoms in a migraine attack and the co-existence of multiple pain disorders, such as temporomandibular disorders and fibromyalgia (Onder et al., 2019; Kleykamp et al., 2021).

The comorbidity in migraine goes beyond chronic pain, and in recent years the associations of migraine and motor disorders are becoming more evident in clinical and translational studies, including depression and Parkinson’s disease (Cervenka et al., 2006; Scher et al., 2014). This is primarily attributed to the imbalance and deficiency in DA D2/D3 receptor (DOR) BP_ND_ in the basal ganglia in migraineurs (DaSilva et al., 2017). Herein, we found that the putamen was the most predictive region in the basal ganglia for migraine regarding DOR D2/D3 BP_ND_ levels. The third was for *μ*-opioid BP_ND_ levels, mainly in its anterior portion, which connects with the large associative regions in the cortex. The putamen is one of the major sites of cortical input into basal ganglia and is commonly activated during pain and pain-related motor responses. There is a D2 receptor activity associated with variability in pain suffering and modulation in both acute and chronic pain disorders. For instance, patients with Parkinson’s disease have increased thermal pain sensitivity. On the contrary, patients with lesions in the putamen have this sensitivity impaired (Lorberboym et al., 2004; Starr et al., 2011), which also extends to psychological pain (Husain et al., 1991).

These findings suggest a neural framework for migraine classification based on µ-opioid and D2/D3 dopamine receptor availability dysfunction, two of the most crucial central endogenous receptor-neurotransmitters mechanisms in the brain. It also contributes to our better understanding of the large association of migraine patients’ pain and discomfort, their increased sensitivity and aversive reactions to environmental stimuli, and co-existing cognitive-motivational disorders, like Parkinson’s disease and depression (Meerwijk et al., 2013).

## Data Availability

The data supporting this study’s findings are available from the corresponding author upon reasonable request. Requests to access the datasets should be directed to AD, adasilva@umich.edu.
