# Supplementary material for: Classifying migraine using PET compressive big data analytics of brain’s μ-opioid and D2/D3 dopamine neurotransmission
Source: Front Pharmacol. 2023 Jun 13;14:1173596. doi: 10.3389/fphar.2023.1173596 (PMC10294712; doi:10.3389/fphar.2023.1173596)
Supplement: Supplementary file 1 [file DataSheet1.docx]

# Frontiers in Pharmacology, 14:1173596, doi:10.3389/fphar.2023.1173596

# Classifying migraine using PET compressive big data analytics of brain’s μ-opioid and D2/D3 dopamine neurotransmission

# by

# Simeone Marino, Hassan Jassar, Dajung J. Kim, Manyoel Lim, Thiago D. Nascimento, Ivo D. Dinov, Robert A. Koeppe, and Alexandre F. DaSilva

# Appendix - Supplementary Material

**Text S1: CBDA results for the Opioid and Dopamine experiments**

Each panel shows a cross-tabulation of observed and predicted classes with associated statistics. P values here are not corrected for any possible minor voxel spatial correlation. The number in square brackets shows the number of features needed to maximize accuracy in each replication.

**Panel A: Opioid Whole Brain Grey Matter Mask - Confusion Matrix and Statistics**

**Replication 1 [20]**

Reference

Prediction 0 1

0 34 1

1 12 91

Accuracy: 0.9058

95% CI: (0.8443, 0.9489)

No Information Rate: 0.6667

P-Value [Acc > NIR]: 4.518e-11

Kappa: 0.7746

Mcnemar's Test P-Value: 0.005546

Sensitivity: 0.7391

Specificity: 0.9891

Pos Pred Value: 0.9714

Neg Pred Value: 0.8835

Prevalence: 0.3333

Detection Rate: 0.2464

Detection Prevalence: 0.2536

Balanced Accuracy: 0.8641

**Replication 2 [7]**

Reference

Prediction 0 1

0 37 0

1 9 92

Accuracy: 0.9348

95% CI: (0.8798, 0.9697)

No Information Rate: 0.6667

P-Value [Acc > NIR]: 4.336e-14

Kappa: 0.8457

Mcnemar's Test P-Value: 0.007661

Sensitivity: 0.8043

Specificity: 1.0000

Pos Pred Value: 1.0000

Neg Pred Value: 0.9109

Prevalence: 0.3333

Detection Rate: 0.2681

Detection Prevalence: 0.2681

Balanced Accuracy: 0.9022

**Replication 3 [11]**

Reference

Prediction 0 1

0 43 0

1 3 92

Accuracy: 0.9783

95% CI: (0.9378, 0.9955)

No Information Rate: 0.6667

P-Value [Acc > NIR]: <2e-16

Kappa: 0.9503

Mcnemar's Test P-Value: 0.2482

Sensitivity: 0.9348

Specificity: 1.0000

Pos Pred Value: 1.0000

Neg Pred Value: 0.9684

Prevalence: 0.3333

Detection Rate: 0.3116

Detection Prevalence: 0.3116

Balanced Accuracy: 0.9674

**Replication 4 [32]**

Reference

Prediction 0 1

0 45 0

1 1 92

Accuracy: 0.9928

95% CI: (0.9603, 0.9998)

No Information Rate: 0.6667

P-Value [Acc > NIR]: <2e-16

Kappa: 0.9836

Mcnemar's Test P-Value: 1

Sensitivity: 0.9783

Specificity: 1.0000

Pos Pred Value: 1.0000

Neg Pred Value: 0.9892

Prevalence: 0.3333

Detection Rate: 0.3261

Detection Prevalence: 0.3261

Balanced Accuracy: 0.9891

**Replication 5 [30]**

Reference

Prediction 0 1

0 41 0

1 5 92

Accuracy: 0.9638

95% CI: (0.9175, 0.9881)

No Information Rate: 0.6667

P-Value [Acc > NIR]: < 2e-16

Kappa: 0.9162

Mcnemar's Test P-Value: 0.07364

Sensitivity: 0.8913

Specificity: 1.0000

Pos Pred Value: 1.0000

Neg Pred Value: 0.9485

Prevalence: 0.3333

Detection Rate: 0.2971

Detection Prevalence: 0.2971

Balanced Accuracy: 0.9457

**Panel B: Dopamine Whole Brain Basal Ganglia Mask - Confusion Matrix and Statistics**

**Replication 1 [3]**

Reference

Prediction 0 1

0 20 0

1 0 40

Accuracy: 1

95% CI: (0.9404, 1)

No Information Rate: 0.6667

P-Value [Acc > NIR]: 2.72e-11

Kappa: 1

Mcnemar's Test P-Value: NA

Sensitivity: 1.0000

Specificity: 1.0000

Pos Pred Value: 1.0000

Neg Pred Value: 1.0000

Prevalence: 0.3333

Detection Rate: 0.3333

Detection Prevalence: 0.3333

Balanced Accuracy: 1.0000

**Replication 2 [4]**

Reference

Prediction 0 1

0 20 0

1 0 40

Accuracy: 1

95% CI: (0.9404, 1)

No Information Rate: 0.6667

P-Value [Acc > NIR]: 2.72e-11

Kappa: 1

Mcnemar's Test P-Value: NA

Sensitivity: 1.0000

Specificity: 1.0000

Pos Pred Value: 1.0000

Neg Pred Value: 1.0000

Prevalence: 0.3333

Detection Rate: 0.3333

Detection Prevalence: 0.3333

Balanced Accuracy: 1.0000

**Replication 3 [17]**

Reference

Prediction 0 1

0 20 0

1 0 40

Accuracy: 1

95% CI: (0.9404, 1)

No Information Rate: 0.6667

P-Value [Acc > NIR]: 2.72e-11

Kappa: 1

Mcnemar's Test P-Value: NA

Sensitivity: 1.0000

Specificity: 1.0000

Pos Pred Value: 1.0000

Neg Pred Value: 1.0000

Prevalence: 0.3333

Detection Rate: 0.3333

Detection Prevalence: 0.3333

Balanced Accuracy: 1.0000

**Replication 4 [3]**

Reference

Prediction 0 1

0 20 0

1 0 40

Accuracy: 1

95% CI: (0.9404, 1)

No Information Rate: 0.6667

P-Value [Acc > NIR]: 2.72e-11

Kappa: 1

Mcnemar's Test P-Value: NA

Sensitivity: 1.0000

Specificity: 1.0000

Pos Pred Value: 1.0000

Neg Pred Value: 1.0000

Prevalence: 0.3333

Detection Rate: 0.3333

Detection Prevalence: 0.3333

Balanced Accuracy: 1.0000

**Replication 5 [3]**

Reference

Prediction 0 1

0 20 0

1 0 40

Accuracy: 1

95% CI: (0.9404, 1)

No Information Rate: 0.6667

P-Value [Acc > NIR]: 2.72e-11

Kappa: 1

Mcnemar's Test P-Value: NA

Sensitivity: 1.0000

Specificity: 1.0000

Pos Pred Valvue: 1.0000

Neg Pred Value: 1.0000

Prevalence: 0.3333

Detection Rate: 0.3333

Detection Prevalence: 0.3333

Balanced Accuracy: 1.0000

**Panel C: Opioid ROIs Grey Matter Mask - Confusion Matrix and Statistics**

**INSULA**

**Replication 1 [8]**

Reference

Prediction 0 1

0 46 0

1 0 92

Accuracy: 1

95% CI: (0.9736, 1)

No Information Rate: 0.6667

P-Value [Acc > NIR]: < 2.2e-16

Kappa: 1

Mcnemar's Test P-Value: NA

Sensitivity: 1.0000

Specificity: 1.0000

Pos Pred Value: 1.0000

Neg Pred Value: 1.0000

Prevalence: 0.3333

Detection Rate: 0.3333

Detection Prevalence: 0.3333

Balanced Accuracy: 1.0000

**Replication 2 [3]**

Reference

Prediction 0 1

0 46 0

1 0 92

Accuracy: 1

95% CI: (0.9736, 1)

No Information Rate: 0.6667

P-Value [Acc > NIR]: < 2.2e-16

Kappa: 1

Mcnemar's Test P-Value: NA

Sensitivity: 1.0000

Specificity: 1.0000

Pos Pred Value: 1.0000

Neg Pred Value: 1.0000

Prevalence: 0.3333

Detection Rate: 0.3333

Detection Prevalence: 0.3333

Balanced Accuracy: 1.0000

**Replication 3 [6]**

Reference

Prediction 0 1

0 46 0

1 0 92

Accuracy: 1

95% CI: (0.9736, 1)

No Information Rate: 0.6667

P-Value [Acc > NIR]: < 2.2e-16

Kappa: 1

Mcnemar's Test P-Value: NA

Sensitivity: 1.0000

Specificity: 1.0000

Pos Pred Value: 1.0000

Neg Pred Value: 1.0000

Prevalence: 0.3333

Detection Rate: 0.3333

Detection Prevalence: 0.3333

Balanced Accuracy: 1.0000

**Replication 4 [3]**

Reference

Prediction 0 1

0 46 0

1 0 92

Accuracy: 1

95% CI: (0.9736, 1)

No Information Rate: 0.6667

P-Value [Acc > NIR]: < 2.2e-16

Kappa: 1

Mcnemar's Test P-Value: NA

Sensitivity: 1.0000

Specificity: 1.0000

Pos Pred Value: 1.0000

Neg Pred Value: 1.0000

Prevalence: 0.3333

Detection Rate: 0.3333

Detection Prevalence: 0.3333

Balanced Accuracy: 1.0000

**Replication 5 [4]**

Reference

Prediction 0 1

0 46 0

1 0 92

Accuracy: 1

95% CI: (0.9736, 1)

No Information Rate: 0.6667

P-Value [Acc > NIR]: < 2.2e-16

Kappa: 1

Mcnemar's Test P-Value: NA

Sensitivity: 1.0000

Specificity: 1.0000

Pos Pred Value: 1.0000

Neg Pred Value: 1.0000

Prevalence: 0.3333

Detection Rate: 0.3333

Detection Prevalence: 0.3333

Balanced Accuracy: 1.0000

**Panel D: Opioid ROIs Grey Matter Mask - Confusion Matrix and Statistics**

**THALAMUS**

**Replication 1 [3]**

Reference

Prediction 0 1

0 46 0

1 0 92

Accuracy: 1

95% CI: (0.9736, 1)

No Information Rate: 0.6667

P-Value [Acc > NIR]: < 2.2e-16

Kappa: 1

Mcnemar's Test P-Value: NA

Sensitivity: 1.0000

Specificity: 1.0000

Pos Pred Value: 1.0000

Neg Pred Value: 1.0000

Prevalence: 0.3333

Detection Rate: 0.3333

Detection Prevalence: 0.3333

Balanced Accuracy: 1.0000

**Replication 2 [4]**

Reference

Prediction 0 1

0 46 0

1 0 92

Accuracy: 1

95% CI: (0.9736, 1)

No Information Rate: 0.6667

P-Value [Acc > NIR]: < 2.2e-16

Kappa: 1

Mcnemar's Test P-Value: NA

Sensitivity: 1.0000

Specificity: 1.0000

Pos Pred Value: 1.0000

Neg Pred Value: 1.0000

Prevalence: 0.3333

Detection Rate: 0.3333

Detection Prevalence: 0.3333

Balanced Accuracy: 1.0000

**Replication 3 [3]**

Reference

Prediction 0 1

0 46 0

1 0 92

Accuracy: 1

95% CI: (0.9736, 1)

No Information Rate: 0.6667

P-Value [Acc > NIR]: < 2.2e-16

Kappa: 1

Mcnemar's Test P-Value: NA

Sensitivity: 1.0000

Specificity: 1.0000

Pos Pred Value: 1.0000

Neg Pred Value: 1.0000

Prevalence: 0.3333

Detection Rate: 0.3333

Detection Prevalence: 0.3333

Balanced Accuracy: 1.0000

**Replication 4 [4]**

Reference

Prediction 0 1

0 46 0

1 0 92

Accuracy: 1

95% CI: (0.9736, 1)

No Information Rate: 0.6667

P-Value [Acc > NIR]: < 2.2e-16

Kappa: 1

Mcnemar's Test P-Value: NA

Sensitivity: 1.0000

Specificity: 1.0000

Pos Pred Value: 1.0000

Neg Pred Value: 1.0000

Prevalence: 0.3333

Detection Rate: 0.3333

Detection Prevalence: 0.3333

Balanced Accuracy: 1.0000

**Replication 5 [3]**

Reference

Prediction 0 1

0 46 0

1 0 92

Accuracy: 1

95% CI: (0.9736, 1)

No Information Rate: 0.6667

P-Value [Acc > NIR]: < 2.2e-16

Kappa: 1

Mcnemar's Test P-Value: NA

Sensitivity: 1.0000

Specificity: 1.0000

Pos Pred Value: 1.0000

Neg Pred Value: 1.0000

Prevalence: 0.3333

Detection Rate: 0.3333

Detection Prevalence: 0.3333

Balanced Accuracy: 1.0000

**Panel E: Opioid ROIs Grey Matter Mask - Confusion Matrix and Statistics**

**PUTAMEN**

**Replication 1 [6]**

Reference

Prediction 0 1

0 46 0

1 0 92

Accuracy: 1

95% CI: (0.9736, 1)

No Information Rate: 0.6667

P-Value [Acc > NIR]: < 2.2e-16

Kappa: 1

Mcnemar's Test P-Value: NA

Sensitivity: 1.0000

Specificity: 1.0000

Pos Pred Value: 1.0000

Neg Pred Value: 1.0000

Prevalence: 0.3333

Detection Rate: 0.3333

Detection Prevalence: 0.3333

Balanced Accuracy: 1.0000

**Replication 2 [3]**

Reference

Prediction 0 1

0 46 0

1 0 92

Accuracy: 1

95% CI: (0.9736, 1)

No Information Rate: 0.6667

P-Value [Acc > NIR]: < 2.2e-16

Kappa: 1

Mcnemar's Test P-Value: NA

Sensitivity: 1.0000

Specificity: 1.0000

Pos Pred Value: 1.0000

Neg Pred Value: 1.0000

Prevalence: 0.3333

Detection Rate: 0.3333

Detection Prevalence: 0.3333

Balanced Accuracy: 1.0000

**Replication 3 [5]**

Reference

Prediction 0 1

0 46 0

1 0 92

Accuracy: 1

95% CI: (0.9736, 1)

No Information Rate: 0.6667

P-Value [Acc > NIR]: < 2.2e-16

Kappa: 1

Mcnemar's Test P-Value: NA

Sensitivity: 1.0000

Specificity: 1.0000

Pos Pred Value: 1.0000

Neg Pred Value: 1.0000

Prevalence: 0.3333

Detection Rate: 0.3333

Detection Prevalence: 0.3333

Balanced Accuracy: 1.0000

**Replication 4 [8]**

Reference

Prediction 0 1

0 46 0

1 0 92

Accuracy: 1

95% CI: (0.9736, 1)

No Information Rate: 0.6667

P-Value [Acc > NIR]: < 2.2e-16

Kappa: 1

Mcnemar's Test P-Value: NA

Sensitivity: 1.0000

Specificity: 1.0000

Pos Pred Value: 1.0000

Neg Pred Value: 1.0000

Prevalence: 0.3333

Detection Rate: 0.3333

Detection Prevalence: 0.3333

Balanced Accuracy: 1.0000

**Replication 5 [4]**

Reference

Prediction 0 1

0 46 0

1 0 92

Accuracy: 1

95% CI: (0.9736, 1)

No Information Rate: 0.6667

P-Value [Acc > NIR]: < 2.2e-16

Kappa: 1

Mcnemar's Test P-Value: NA

Sensitivity: 1.0000

Specificity: 1.0000

Pos Pred Value: 1.0000

Neg Pred Value: 1.0000

Prevalence: 0.3333

Detection Rate: 0.3333

Detection Prevalence: 0.3333

Balanced Accuracy: 1.0000

'Positive' Class: 0

**Panel F: Dopamine ROI Basal Ganglia Mask - Confusion Matrix and Statistics**

**PUTAMEN**

**Replication 1 [6]**

Reference

Prediction 0 1

0 20 0

1 0 40

Accuracy: 1

95% CI: (0.9404, 1)

No Information Rate: 0.6667

P-Value [Acc > NIR]: 2.72e-11

Kappa: 1

Mcnemar's Test P-Value: NA

Sensitivity: 1.0000

Specificity: 1.0000

Pos Pred Value: 1.0000

Neg Pred Value: 1.0000

Prevalence: 0.3333

Detection Rate: 0.3333

Detection Prevalence: 0.3333

Balanced Accuracy: 1.0000

**Replication 2 [3]**

Reference

Prediction 0 1

0 20 0

1 0 40

Accuracy: 1

95% CI: (0.9404, 1)

No Information Rate: 0.6667

P-Value [Acc > NIR]: 2.72e-11

Kappa: 1

Mcnemar's Test P-Value: NA

Sensitivity: 1.0000

Specificity: 1.0000

Pos Pred Value: 1.0000

Neg Pred Value: 1.0000

Prevalence: 0.3333

Detection Rate: 0.3333

Detection Prevalence: 0.3333

Balanced Accuracy: 1.0000

**Replication 3 [5]**

Reference

Prediction 0 1

0 20 0

1 0 40

Accuracy: 1

95% CI: (0.9404, 1)

No Information Rate: 0.6667

P-Value [Acc > NIR]: 2.72e-11

Kappa: 1

Mcnemar's Test P-Value: NA

Sensitivity: 1.0000

Specificity: 1.0000

Pos Pred Value: 1.0000

Neg Pred Value: 1.0000

Prevalence: 0.3333

Detection Rate: 0.3333

Detection Prevalence: 0.3333

Balanced Accuracy: 1.0000

**Replication 4 [11]**

Reference

Prediction 0 1

0 20 0

1 0 40

Accuracy: 1

95% CI: (0.9404, 1)

No Information Rate: 0.6667

P-Value [Acc > NIR]: 2.72e-11

Kappa: 1

Mcnemar's Test P-Value: NA

Sensitivity: 1.0000

Specificity: 1.0000

Pos Pred Value: 1.0000

Neg Pred Value: 1.0000

Prevalence: 0.3333

Detection Rate: 0.3333

Detection Prevalence: 0.3333

Balanced Accuracy: 1.0000

**Replication 5 [11]**

Reference

Prediction 0 1

0 20 0

1 0 40

Accuracy: 1

95% CI: (0.9404, 1)

No Information Rate: 0.6667

P-Value [Acc > NIR]: 2.72e-11

Kappa: 1

Mcnemar's Test P-Value: NA

Sensitivity: 1.0000

Specificity: 1.0000

Pos Pred Value: 1.0000

Neg Pred Value: 1.0000

Prevalence: 0.3333

Detection Rate: 0.3333

Detection Prevalence: 0.3333

Balanced Accuracy: 1.0000

**Supplementary Text S2 – Top predictive voxels statistics**

|  | **Grey Matter Mask – ROIs**  **(top 50 predictive voxels across 5 CBDA experiments)** | | |
| --- | --- | --- | --- |
|  | Insula [voxels] | Thalamus [voxels] | Putamen [voxels] |
| Migraine | 1.9392 | 2.303524 | 2.330997 |
| Health | 1.975582 | 2.460420 | 2.431706 |
| Significance | NS | p<1e-5 | p<0.011 |
| Min-Max | [0.88569 , 3.26410] | [0.91005, 5.37850] | [0.92291, 5.65140] |

| **Basal Ganglia Mask - Whole Mask and Putamen**  **(top 50 predictive voxels across 5 CBDA experiments)** | | |
| --- | --- | --- |
|  | Basal Ganglia mask  [all voxels] | Putamen [just Putamen voxels] |
| Migraine | 2.984937 | 2.769699 |
| Health | 2.964039 | 2.783846 |
| Significance | p<0.03655 | NS |
| Min-Max | [1.3085,5.0166] | [1.1655,4.797] |

**Supplementary Text S3 – CBDA External Cross Validation**

Due to the limited number of subjects, the results showcased so far in the study are based on internal cross validation analyses. As a proof of concept, we showcase here a tentative CBDA external cross validation experiment performed on the Grey Matter mask for the whole brain BP_ND_ μ-opioid Carfentanil data. Due to the low number of subjects, it was not feasible to obtain a large enough and balanced subsample for the Dopamine BP_ND_ dataset. In order to achieve meaningful results, we used a 20%-80% split between validation and training set. Based on the demographics (23 healthy, 46 migraine) and since each subject has 2 PET images (early and late), we selected a total of 24 images, representing 4 healthy (2 males, 2 females, 8 PET images [early/late]) and 8 migraine (4 males, 4 females, 16 PET images [early/late]) subjects. Similarly to our main study, we then performed 5 CBDA replications using the 80% of the subjects for training and the 20% as validation, with the Grey Matter mask data (Whole Brain).

We then merged the top predictive voxels across the 5 replications and used the subsets of the the voxels returned from the best predictive model in each replication, for a total of 170 unique voxels. A list of top predictive ROIs is given below (confirming our main results with Insula, Thalamus and Putamen as the top 3 predictive ROIs). Tables below also show the predictions details (accuracy, sensitivity, specificity, likelihoods and MNI coordinates).

**Confusion Matrix and Statistics**

| **Accuracy: 0.9583** | 95%, CI:(0.7888,0.9989) | No. Information Rate: 0.6667  P-Value,[Acc,>,NIR]: 0.0007722 | Kappa: 0.9032 |
| --- | --- | --- | --- |
| Sensitivity: 0.8750 | Specificity: 1.0000 | Mcnemar's,Test,P-Value: 1.0 | Pos.Pred.Value: 1.0000 |
| Prevalence: 0.3333 | Detection,Rate: 0.2917 | Neg. Pred. Value: 0.9412 | 'Positive' Class : 0 (Healthy) |
| Detection Prevalence: 0.2917 | | Balanced,Accuracy: 0.9375 |  |

This is the breakdown of the true/false positives/negatives

|  | Reference | |
| --- | --- | --- |
| Prediction | 0 | 1 |
| 0 | 7 | 0 |
| 1 | 1 | 16 |

**This is the list of the 24 images (12 subjects, 4 healthy vs 8 migraine) with the correspondent likelihoods and predictions. Outcome represents the true class. Here we run training on 80% and validation on the 20%, only using 170 voxels.**

| **ImageID** | **Likelihood** | **Prediction** | **Outcome** |
| --- | --- | --- | --- |
| 1 | 0.465064 | 0 | 0 |
| 2 | 0.279626 | 0 | 0 |
| 3 | 0.386487 | 0 | 0 |
| 4 | 0.483287 | 0 | 0 |
| 37 | 0.283809 | 0 | 0 |
| 38 | 0.310616 | 0 | 0 |
| 39 | 0.840784 | 1 | 0 |
| 40 | 0.499565 | 0 | 0 |
| 49 | 0.661132 | 1 | 1 |
| 50 | 0.831939 | 1 | 1 |
| 51 | 0.804819 | 1 | 1 |
| 52 | 0.834866 | 1 | 1 |
| 53 | 0.733485 | 1 | 1 |
| 54 | 0.780606 | 1 | 1 |
| 55 | 0.754812 | 1 | 1 |
| 56 | 0.769519 | 1 | 1 |
| 121 | 0.678761 | 1 | 1 |
| 122 | 0.726084 | 1 | 1 |
| 125 | 0.739694 | 1 | 1 |
| 126 | 0.791001 | 1 | 1 |
| 127 | 0.733726 | 1 | 1 |
| 128 | 0.755827 | 1 | 1 |
| 129 | 0.893018 | 1 | 1 |
| 130 | 0.941489 | 1 | 1 |

**This is the top predictive ROI ranking (calculated by ranking the frequency of top predictive voxels belonging to a specific ROI)**

| **ROI** | **#voxels** | **%voxels** |
| --- | --- | --- |
| 'Insula' | 31 | 18.3432 |
| 'Putamen' | 28 | 16.5680 |
| 'Thalamus' | 26 | 15.3846 |
| 'Caudate' | 11 | 6.5089 |
| 'Amygdala' | 9 | 5.3254 |
| 'Anterior_Cingulate' | 9 | 5.3254 |
| 'Frontal_Med_Orb' | 9 | 5.3254 |
| 'Hippocampus' | 8 | 4.7337 |
| 'Pallidum' | 8 | 4.7337 |
| 'Claustrum' | 5 | 2.9586 |
| 'Subcallosal_Gyrus' | 5 | 2.9586 |
| 'Rectus' | 4 | 2.3669 |
| 'Temporal_Pole_Mid' | 3 | 1.7751 |
| 'Caudate_Head' | 2 | 1.1834 |
| 'Hypothalamus' | 2 | 1.1834 |
| 'Parahippocampa_Gyrus' | 2 | 1.1834 |
| 'Temporal_Inf' | 2 | 1.1834 |
| 'Cingulate_Gyrus' | 1 | 0.5917 |
| 'Lateral_Globus_Pallidus' | 1 | 0.5917 |
| 'ParaHippocampal' | 1 | 0.5917 |
| 'Temporal_Pole_Sup' | 1 | 0.5917 |
| 'Ventral_Anterior_Nucleus' | 1 | 0.5917 |

**Top predictive voxels returned by CBDA across 5 replications (subset of 170 out of ~500K) listed by:**

1. **Matlab coordinates (1D array)**
2. **MNI coordinates (conversion from the 1D array into 3D coordinates [X,Y,Z] ROI label description.**

**Note: the order is solely based on the Matlab 1D array index (i.e., ascending order)**

| # | Matlab | **X** | **Y** | **Z** | **ROI label** |
| --- | --- | --- | --- | --- | --- |
| 1 | 42046 | 44 | 2 | -40 | 'Temporal_Inf_R_aal' |
| 2 | 49827 | -34 | 8 | -38 | 'Temporal_Inf_L_aal' |
| 3 | 72380 | 48 | 10 | -32 | 'Temporal_Pole_Mid_R_aal' |
| 4 | 72381 | 46 | 10 | -32 | 'Temporal_Pole_Mid_R_aal' |
| 5 | 72425 | -42 | 10 | -32 | 'Temporal_Pole_Mid_L_aal' |
| 6 | 101860 | 22 | -4 | -24 | 'ParaHippocampal_R_aal' |
| 7 | 109309 | -24 | -6 | -22 | 'Hippocampus_L_aal' |
| 8 | 109362 | 28 | -4 | -22 | 'Hippocampus_R_aal' |
| 9 | 109444 | 22 | -2 | -22 | 'Hippocampus_R_aal' |
| 10 | 109466 | -22 | -2 | -22 | 'Amygdala_L_aal' |
| 11 | 109470 | -30 | -2 | -22 | 'Amygdala_L_aal' |
| 12 | 116789 | 26 | -6 | -20 | 'Hippocampus_R_aal' |
| 13 | 116811 | -18 | -6 | -20 | 'Hippocampus_L_aal' |
| 14 | 116815 | -26 | -6 | -20 | 'Hippocampus_L_aal' |
| 15 | 116866 | 30 | -4 | -20 | 'Amygdala_R_aal' |
| 16 | 117130 | -24 | 2 | -20 | 'Amygdala_L_aal' |
| 17 | 117290 | -28 | 6 | -20 | 'Temporal_Pole_Sup_L_aal' |
| 18 | 117576 | 32 | 14 | -20 | 'Insula_R_aal' |
| 19 | 117607 | -30 | 14 | -20 | 'Insula_L_aal' |
| 20 | 124237 | -18 | -8 | -18 | 'Hippocampus_L_aal' |
| 21 | 124294 | 26 | -6 | -18 | 'Hippocampus_R_aal' |
| 22 | 124320 | -26 | -6 | -18 | 'Amygdala_L_aal' |
| 23 | 124451 | 28 | -2 | -18 | 'Amygdala_R_aal' |
| 24 | 124635 | -24 | 2 | -18 | 'Amygdala_L_aal' |
| 25 | 124713 | -22 | 4 | -18 | 'Parahippocampa_Gyrus' |
| 26 | 125004 | 28 | 12 | -18 | 'Insula_R_aal' |
| 27 | 125569 | 4 | 26 | -18 | 'Rectus_R_aal' |
| 28 | 126046 | -2 | 38 | -18 | 'Rectus_L_aal' |
| 29 | 131798 | 28 | -6 | -16 | 'Amygdala_R_aal' |
| 30 | 132275 | 22 | 6 | -16 | 'Amygdala_R_aal' |
| 31 | 132539 | -32 | 12 | -16 | 'Insula_L_aal' |
| 32 | 132744 | 32 | 18 | -16 | 'Insula_R_aal' |
| 33 | 133075 | 2 | 26 | -16 | 'Rectus_L_aal' |
| 34 | 139716 | -8 | 4 | -14 | 'Subcallosal_Gyrus' |
| 35 | 139725 | -26 | 4 | -14 | 'Parahippocampa_Gyrus' |
| 36 | 139803 | -24 | 6 | -14 | 'Subcallosal_Gyrus' |
| 37 | 139946 | 6 | 10 | -14 | 'Subcallosal_Gyrus' |
| 38 | 140738 | 2 | 30 | -14 | 'Frontal_Med_Orb_L_aal' |
| 39 | 140898 | -2 | 34 | -14 | 'Frontal_Med_Orb_L_aal' |
| 40 | 140974 | 4 | 36 | -14 | 'Frontal_Med_Orb_R_aal' |
| 41 | 147286 | 20 | 6 | -12 | 'Putamen' |
| 42 | 147303 | -14 | 6 | -12 | 'Putamen' |
| 43 | 147305 | -18 | 6 | -12 | 'Putamen' |
| 44 | 147381 | -12 | 8 | -12 | 'Caudate_L_aal' |
| 45 | 147609 | 6 | 14 | -12 | 'Subcallosal_Gyrus' |
| 46 | 147611 | 2 | 14 | -12 | 'Subcallosal_Gyrus' |
| 47 | 147762 | 16 | 18 | -12 | 'Rectus_R_aal' |
| 48 | 147788 | -36 | 18 | -12 | 'Insula_L_aal' |
| 49 | 148322 | 2 | 32 | -12 | 'Frontal_Med_Orb_R_aal' |
| 50 | 148404 | -4 | 34 | -12 | 'Frontal_Med_Orb_L_aal' |
| 51 | 154871 | 18 | 8 | -10 | 'Putamen_R_aal' |
| 52 | 154876 | 8 | 8 | -10 | 'Caudate_R_aal' |
| 53 | 154881 | -2 | 8 | -10 | 'Anterior_Cingulate' |
| 54 | 154949 | 20 | 10 | -10 | 'Putamen_R_aal' |
| 55 | 155019 | 38 | 12 | -10 | 'Insula_R_aal' |
| 56 | 155124 | -14 | 14 | -10 | 'Putamen_L_aal' |
| 57 | 155135 | -36 | 14 | -10 | 'Insula_L_aal' |
| 58 | 155177 | 38 | 16 | -10 | 'Insula_R_aal' |
| 59 | 155290 | -30 | 18 | -10 | 'Insula_L_aal' |
| 60 | 155294 | -38 | 18 | -10 | 'Insula_L_aal' |
| 61 | 155367 | -26 | 20 | -10 | 'Insula_L_aal' |
| 62 | 155905 | 4 | 34 | -10 | 'Frontal_Med_Orb_R_aal' |
| 63 | 155909 | -4 | 34 | -10 | 'Anterior_Cingulate' |
| 64 | 155988 | -4 | 36 | -10 | 'Frontal_Med_Orb_L_aal' |
| 65 | 156300 | 4 | 44 | -10 | 'Frontal_Med_Orb_R_aal' |
| 66 | 161988 | 4 | -2 | -8 | 'Hypothalamus' |
| 67 | 162217 | 20 | 4 | -8 | 'Putamen' |
| 68 | 162455 | 18 | 10 | -8 | 'Putamen_R_aal' |
| 69 | 162616 | 12 | 14 | -8 | 'Caudate_R_aal' |
| 70 | 162761 | 38 | 18 | -8 | 'Insula_R_aal' |
| 71 | 169553 | 42 | 0 | -6 | 'Insula_R_aal' |
| 72 | 169583 | -18 | 0 | -6 | 'Pallidum_L_aal' |
| 73 | 169591 | -34 | 0 | -6 | 'Claustrum' |
| 74 | 169647 | 12 | 2 | -6 | 'Pallidum_R_aal' |
| 75 | 169725 | 14 | 4 | -6 | 'Pallidum_R_aal' |
| 76 | 169738 | -12 | 4 | -6 | 'Pallidum_L_aal' |
| 77 | 169810 | 2 | 6 | -6 | 'Anterior_Cingulate' |
| 78 | 169874 | 32 | 8 | -6 | 'Putamen_R_aal' |
| 79 | 169881 | 18 | 8 | -6 | 'Putamen_R_aal' |
| 80 | 170029 | 38 | 12 | -6 | 'Insula_R_aal' |
| 81 | 170068 | -40 | 12 | -6 | 'Insula_L_aal' |
| 82 | 170369 | -10 | 20 | -6 | 'Caudate_L_aal' |
| 83 | 170378 | -28 | 20 | -6 | 'Insula_L_aal' |
| 84 | 176917 | 8 | -4 | -4 | 'Hypothalamus' |
| 85 | 177165 | -14 | 2 | -4 | 'Pallidum_L_aal' |
| 86 | 177170 | -24 | 2 | -4 | 'Putamen_L_aal' |
| 87 | 177247 | -20 | 4 | -4 | 'Pallidum_L_aal' |
| 88 | 177294 | 44 | 6 | -4 | 'Insula_R_aal' |
| 89 | 177337 | -42 | 6 | -4 | 'Insula_L_aal' |
| 90 | 177391 | 8 | 8 | -4 | 'Caudate_R_aal' |
| 91 | 177465 | 18 | 10 | -4 | 'Putamen_R_aal' |
| 92 | 177615 | 34 | 14 | -4 | 'Insula' |
| 93 | 177624 | 16 | 14 | -4 | 'Putamen' |
| 94 | 177648 | -32 | 14 | -4 | 'Insula_L_aal' |
| 95 | 177689 | 44 | 16 | -4 | 'Insula_R_aal' |
| 96 | 177691 | 40 | 16 | -4 | 'Insula_R_aal' |
| 97 | 177800 | -20 | 18 | -4 | 'Putamen_L_aal' |
| 98 | 177803 | -26 | 18 | -4 | 'Claustrum' |
| 99 | 177805 | -30 | 18 | -4 | 'Insula_L_aal' |
| 100 | 177866 | 6 | 20 | -4 | 'Anterior_Cingulate' |
| 101 | 178737 | 2 | 42 | -4 | 'Frontal_Med_Orb_R_aal' |
| 102 | 178819 | -4 | 44 | -4 | 'Anterior_Cingulate' |
| 103 | 183405 | -12 | -30 | -2 | 'Thalamus' |
| 104 | 183550 | 14 | -26 | -2 | 'Thalamus_R_aal' |
| 105 | 183551 | 12 | -26 | -2 | 'Thalamus_R_aal' |
| 106 | 183796 | -4 | -20 | -2 | 'Thalamus_L_aal' |
| 107 | 184329 | 36 | -6 | -2 | 'Putamen_R_aal' |
| 108 | 184759 | -34 | 4 | -2 | 'Claustrum' |
| 109 | 184838 | -34 | 6 | -2 | 'Claustrum' |
| 110 | 184887 | 26 | 8 | -2 | 'Putamen_R_aal' |
| 111 | 184916 | -32 | 8 | -2 | 'Claustrum' |
| 112 | 185046 | 24 | 12 | -2 | 'Putamen_R_aal' |
| 113 | 185056 | 4 | 12 | -2 | 'Caudate_Head' |
| 114 | 185195 | 42 | 16 | -2 | 'Insula_R_aal' |
| 115 | 185282 | 26 | 18 | -2 | 'Putamen_R_aal' |
| 116 | 185284 | 22 | 18 | -2 | 'Putamen_R_aal' |
| 117 | 185305 | -20 | 18 | -2 | 'Putamen_L_aal' |
| 118 | 185309 | -28 | 18 | -2 | 'Insula' |
| 119 | 186403 | -4 | 46 | -2 | 'Anterior_Cingulate' |
| 120 | 186404 | -6 | 46 | -2 | 'Anterior_Cingulate' |
| 121 | 190975 | 16 | -28 | 0 | 'Thalamus_R_aal' |
| 122 | 190988 | -10 | -28 | 0 | 'Thalamus_L_aal' |
| 123 | 190989 | -12 | -28 | 0 | 'Thalamus_L_aal' |
| 124 | 191213 | 14 | -22 | 0 | 'Thalamus_R_aal' |
| 125 | 191451 | 12 | -16 | 0 | 'Thalamus_R_aal' |
| 126 | 191541 | -10 | -14 | 0 | 'Thalamus_L_aal' |
| 127 | 191620 | -10 | -12 | 0 | 'Thalamus_L_aal' |
| 128 | 191910 | 42 | -4 | 0 | 'Insula_R_aal' |
| 129 | 192241 | 12 | 4 | 0 | 'Lateral_Globus_Pallidus' |
| 130 | 192253 | -12 | 4 | 0 | 'Pallidum_L_aal' |
| 131 | 192258 | -22 | 4 | 0 | 'Putamen_L_aal' |
| 132 | 192413 | -16 | 8 | 0 | 'Pallidum_L_aal' |
| 133 | 192574 | -22 | 12 | 0 | 'Putamen_L_aal' |
| 134 | 192639 | 6 | 14 | 0 | 'Caudate_R_aal' |
| 135 | 192738 | -34 | 16 | 0 | 'Insula_L_aal' |
| 136 | 193666 | 6 | 40 | 0 | 'Anterior_Cingulate' |
| 137 | 198336 | -12 | -32 | 2 | 'Thalamus_L_aal' |
| 138 | 198718 | 14 | -22 | 2 | 'Thalamus_R_aal' |
| 139 | 198727 | -4 | -22 | 2 | 'Thalamus_L_aal' |
| 140 | 199039 | 4 | -14 | 2 | 'Thalamus_R_aal' |
| 141 | 199217 | -36 | -10 | 2 | 'Insula_L_aal' |
| 142 | 199283 | -10 | -8 | 2 | 'Thalamus_L_aal' |
| 143 | 199377 | -40 | -6 | 2 | 'Insula_L_aal' |
| 144 | 199378 | -42 | -6 | 2 | 'Insula' |
| 145 | 199841 | -20 | 6 | 2 | 'Putamen_L_aal' |
| 146 | 199897 | 26 | 8 | 2 | 'Putamen_R_aal' |
| 147 | 199905 | 10 | 8 | 2 | 'Caudate_R_aal' |
| 148 | 200154 | -14 | 14 | 2 | 'Caudate_Head' |
| 149 | 200157 | -20 | 14 | 2 | 'Putamen_L_aal' |
| 150 | 200219 | 14 | 16 | 2 | 'Caudate_R_aal' |
| 151 | 205918 | -8 | -30 | 4 | 'Thalamus_L_aal' |
| 152 | 206147 | 8 | -24 | 4 | 'Thalamus_R_aal' |
| 153 | 206382 | 12 | -18 | 4 | 'Thalamus_R_aal' |
| 154 | 206709 | -10 | -10 | 4 | 'Thalamus_L_aal' |
| 155 | 206858 | 8 | -6 | 4 | 'Thalamus_R_aal' |
| 156 | 207113 | -28 | 0 | 4 | 'Putamen_L_aal' |
| 157 | 207235 | 44 | 4 | 4 | 'Insula_R_aal' |
| 158 | 207478 | 32 | 10 | 4 | 'Putamen_R_aal' |
| 159 | 207504 | -20 | 10 | 4 | 'Putamen_L_aal' |
| 160 | 207881 | 16 | 20 | 4 | 'Caudate_R_aal' |
| 161 | 213585 | -16 | -26 | 6 | 'Thalamus_L_aal' |
| 162 | 214210 | -2 | -10 | 6 | 'Thalamus_L_aal' |
| 163 | 214993 | 12 | 10 | 6 | 'Caudate_R_aal' |
| 164 | 215150 | 14 | 14 | 6 | 'Caudate_R_aal' |
| 165 | 221870 | 4 | -6 | 8 | 'Thalamus' |
| 166 | 223608 | 4 | 38 | 8 | 'Anterior_Cingulate' |
| 167 | 229221 | -4 | -10 | 10 | 'Thalamus_L_aal' |
| 168 | 229375 | 4 | -6 | 10 | 'Thalamus' |
| 169 | 229451 | 10 | -4 | 10 | 'Ventral_Anterior_Nucleus' |
| 170 | 342190 | -2 | 0 | 40 | 'Cingulate_Gyrus' |

**Supplementary Text S4 – CBDA Internal Cross Validation – Interictal Only**

As a proof of concept, similarly to the external cross validation effort, we showcase here CBDA results on the interictal patients and images only (Grey Matter mask for the whole brain BP_ND_ μ-opioid Carfentanil data). Based on **Table 1**, we included 108 images from 31 patients and 23 HC for the CBDA protocol (all the Ictal patients/images).

Similarly to our main study, we then performed our CBDA analysis.

A list of top predictive ROIs is given below (confirming our main results with Insula, Thalamus and Putamen as the top 3 predictive ROIs). Tables below also show the predictions details (accuracy, sensitivity, specificity, likelihoods and MNI coordinates).

**Confusion Matrix and Statistics**

| **Accuracy: 1** | 95%, CI:(0.967,1) | No. Information Rate: 0.5818  P-Value,[Acc,>,NIR]: 2.2e-16 | Kappa: 1 |
| --- | --- | --- | --- |
| Sensitivity: 1 | Specificity: 1 | Mcnemar's,Test,P-Value: NA | Pos.Pred.Value: 1.0000 |
| Prevalence: 0.4182 | Detection,Rate: 0. 4182 | Neg. Pred. Value: 1 | 'Positive' Class : 0 (Healthy) |
| Detection Prevalence: 0.4182 | | Balanced,Accuracy: 1 |  |

This is the breakdown of the true/false positives/negatives

|  | Reference | |
| --- | --- | --- |
| Prediction | 0 | 1 |
| 0 | 46 | 0 |
| 1 | 0 | 64 |

**This is the top predictive ROI ranking (calculated by ranking the frequency of top predictive voxels belonging to a specific ROI)**

| **ROI** | **#voxels** | **%voxels** |
| --- | --- | --- |
| 'Insula' | 9 | 18.75 |
| 'Putamen' | 9 | 18.75 |
| 'Thalamus' | 6 | 12.5 |
| 'Caudate' | 6 | 12.5 |
| 'Amygdala' | 6 | 12.5 |
